# Supplementary material for: Examination of two different proteasome inhibitors in reactivating mutant human cystathionine β-synthase in mice
Source: PLoS One. 2023 Jun 15;18(6):e0286550. doi: 10.1371/journal.pone.0286550 (PMC10270616; doi:10.1371/journal.pone.0286550)
Supplement: S2 File — File shows complete image data for all Western blots, as well as molecular weight migration markers. (PDF) [file pone.0286550.s002.pdf]

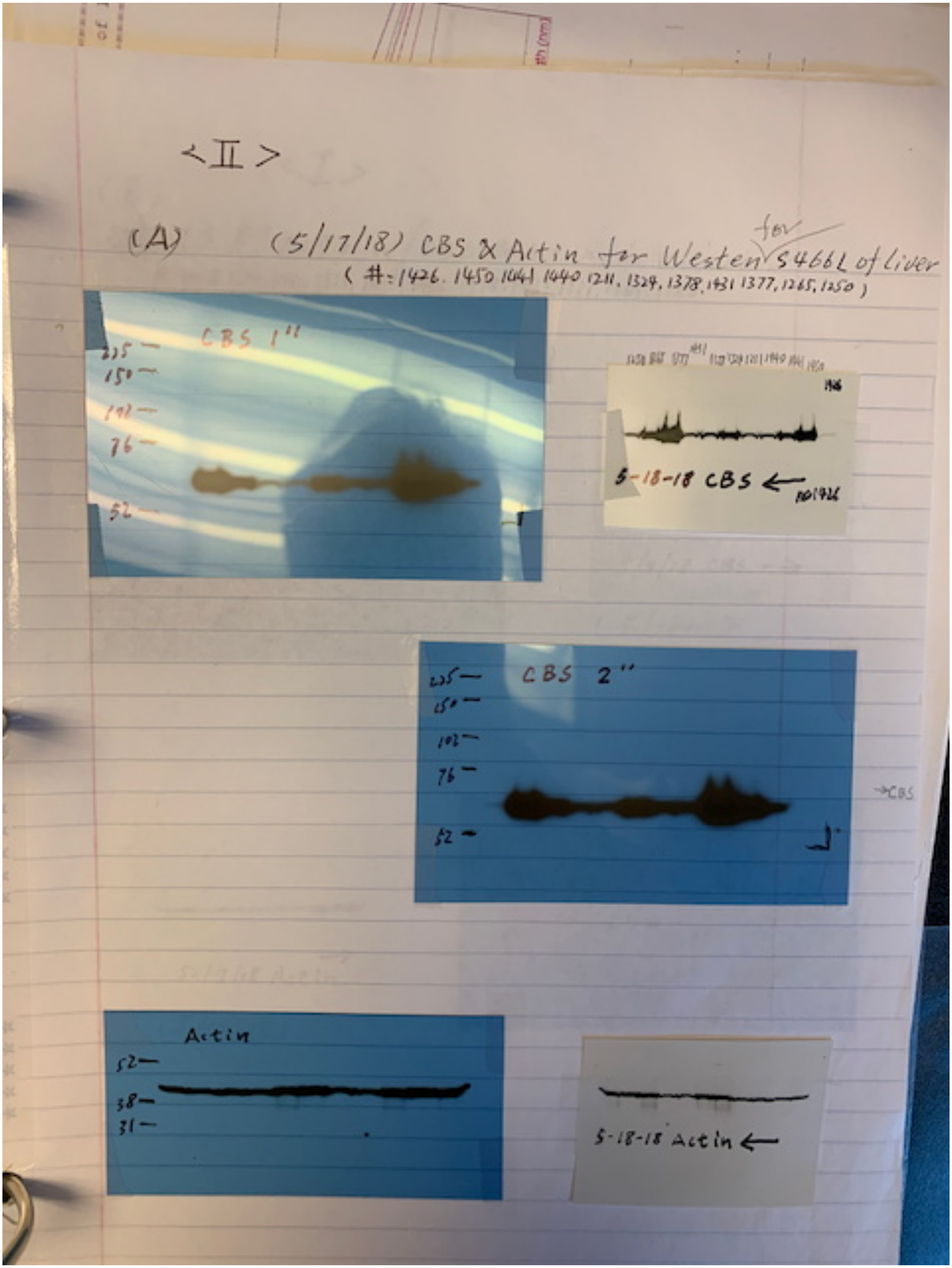

Figure 1 Raw Data. Film and Imager print.

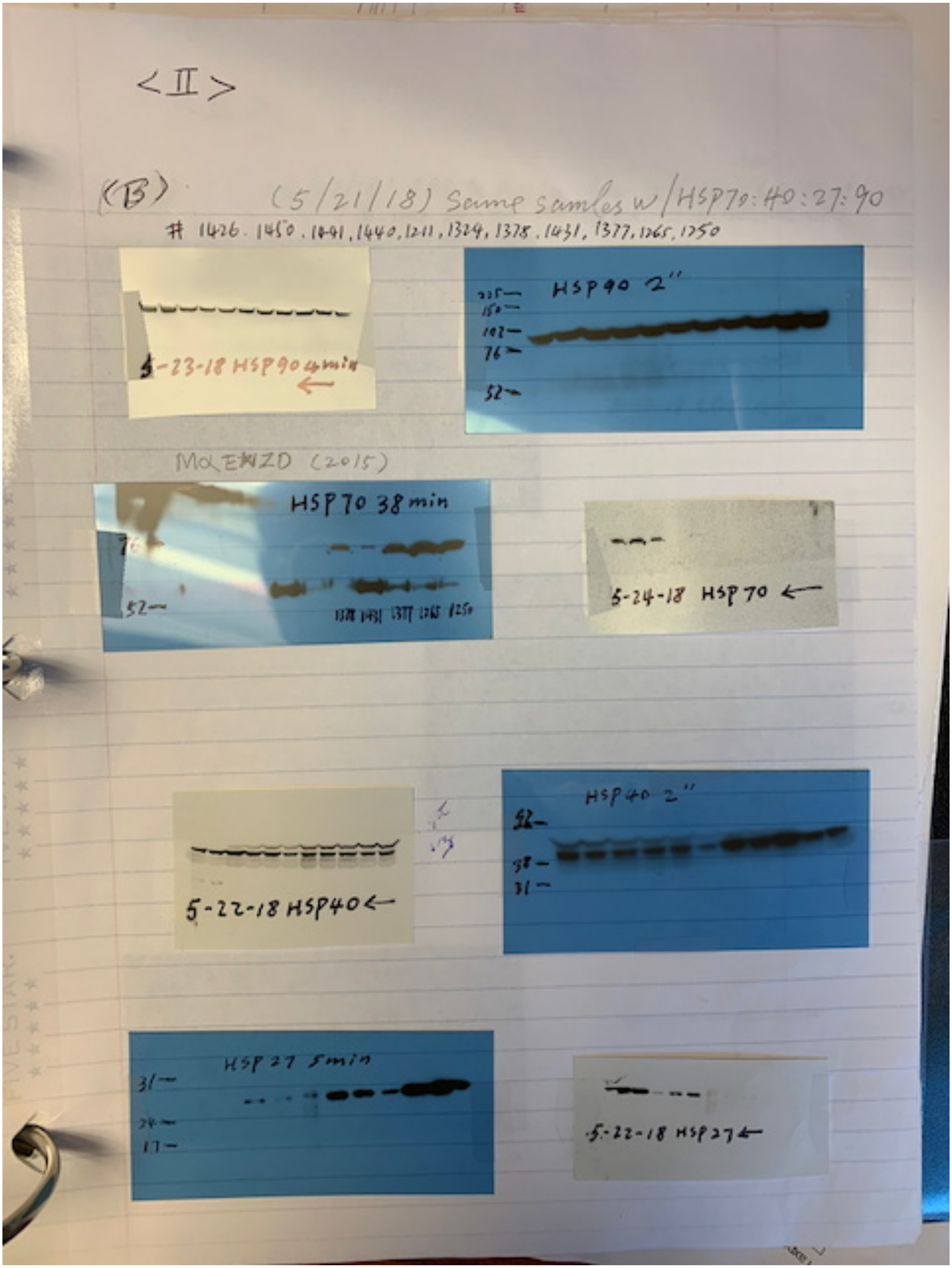

Figure 1 Raw Data. Film and Imager print.

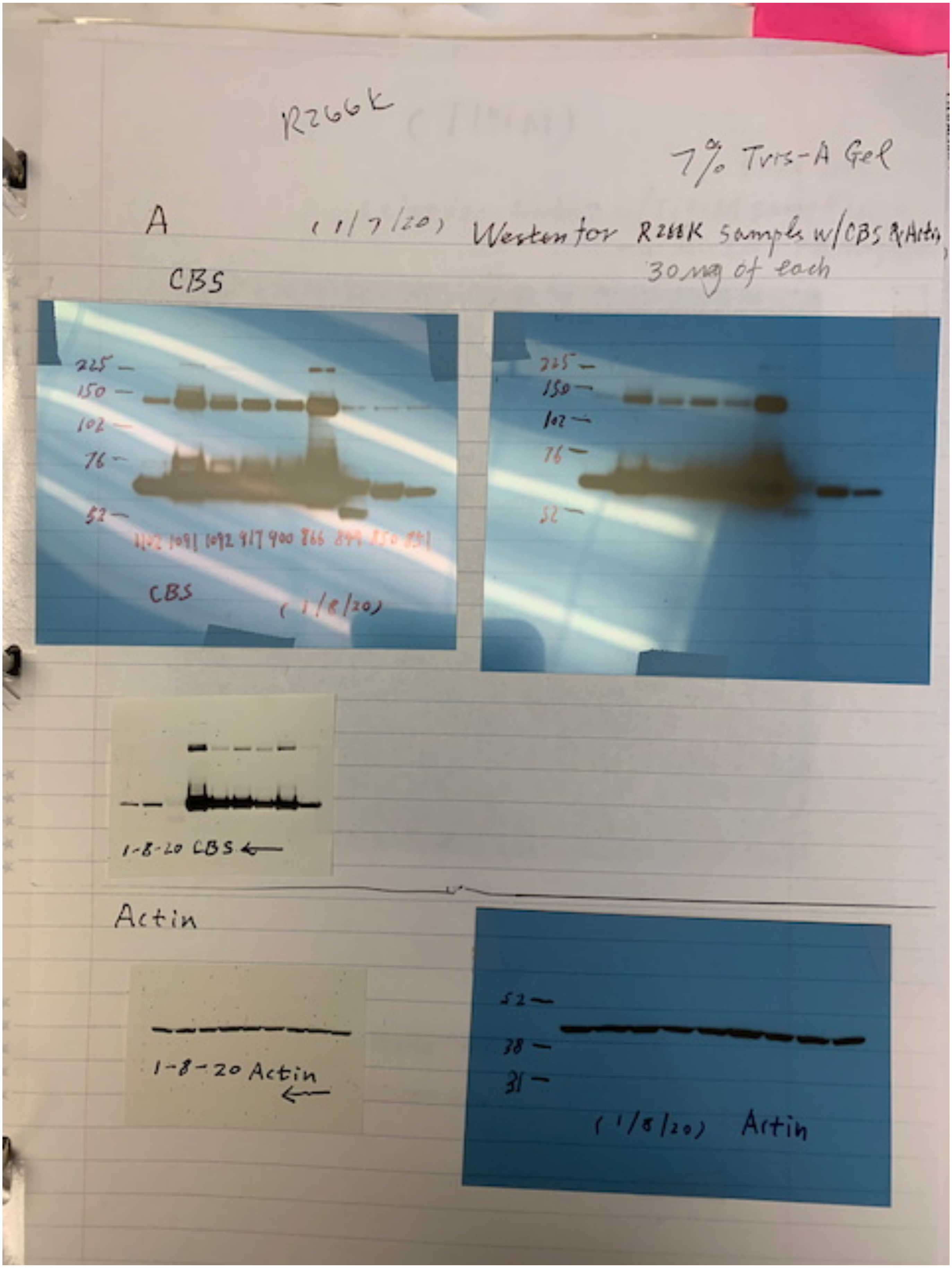

Figure 2a Raw Data

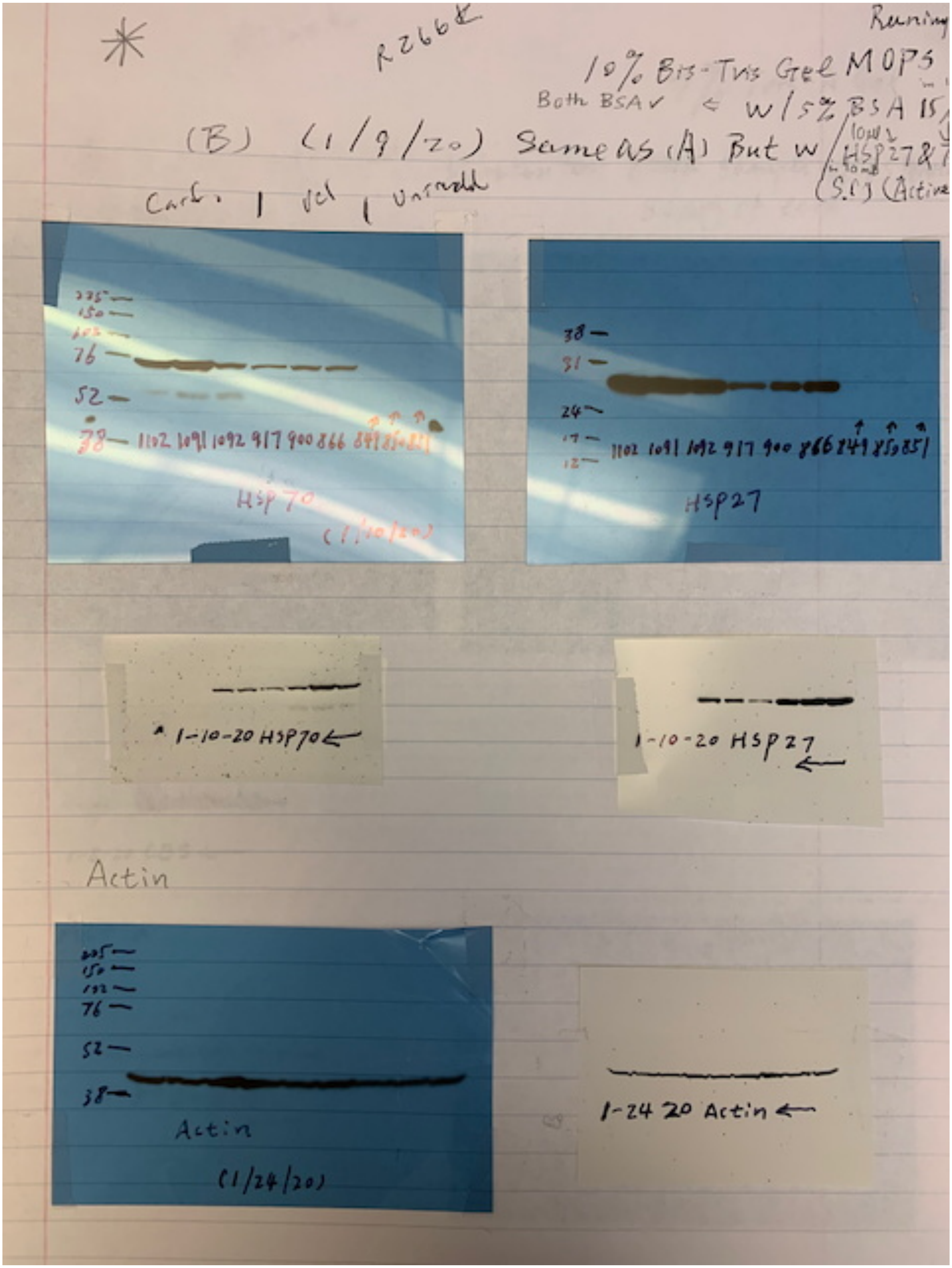

Figure 2a Raw data

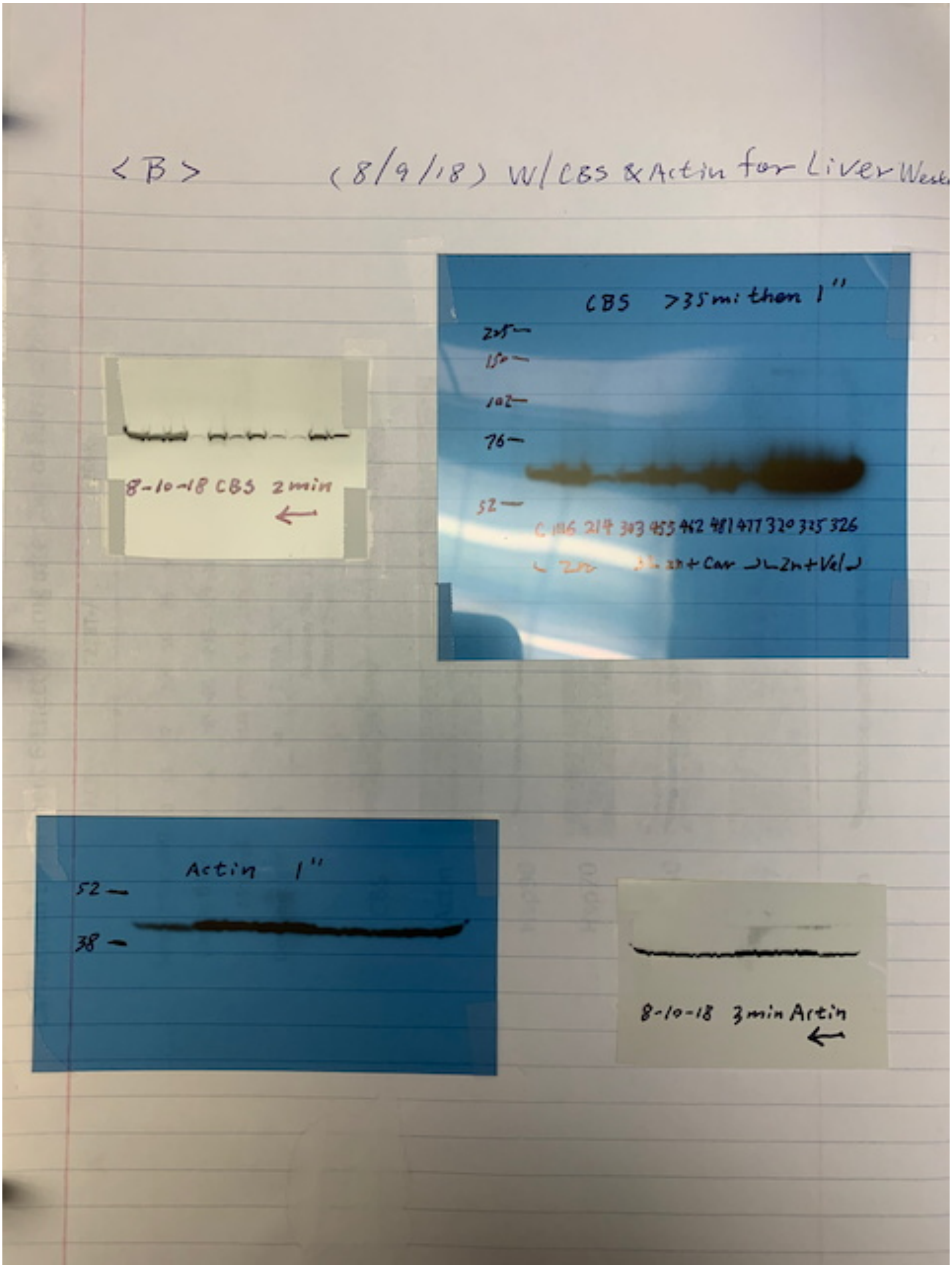

Figure 2B. Raw data.

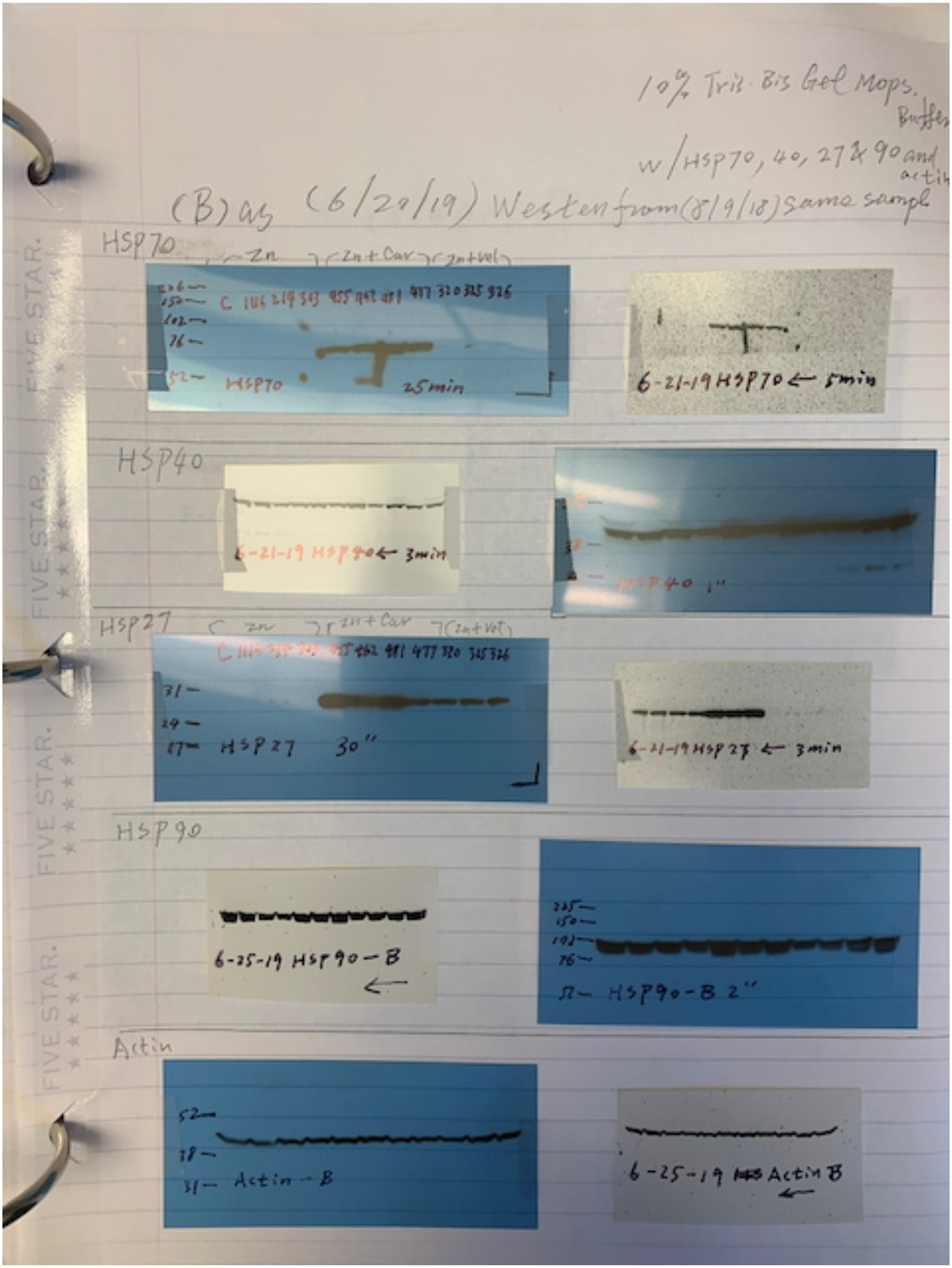

Figure 2B. Raw Data.

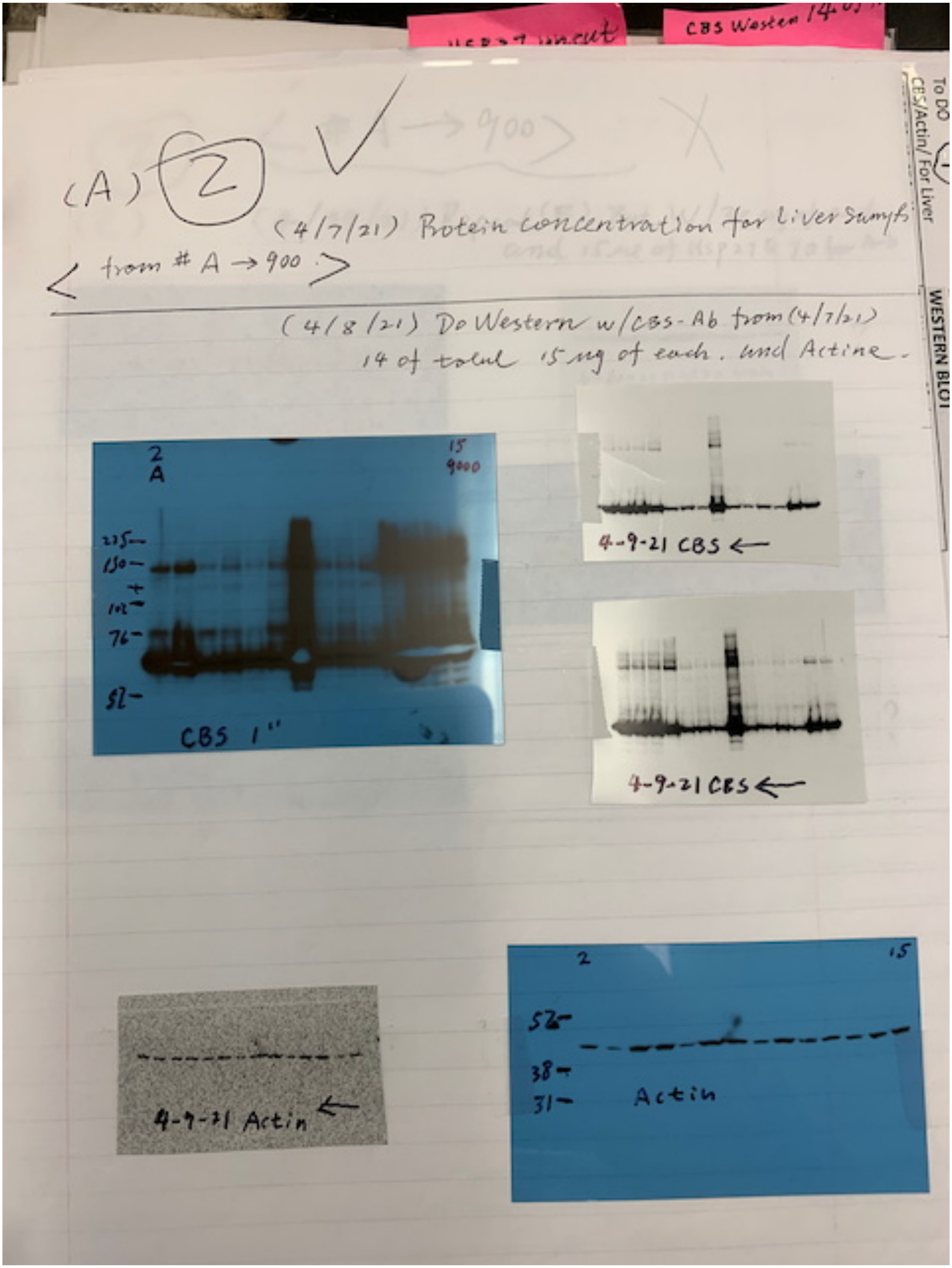

Figure 2c data

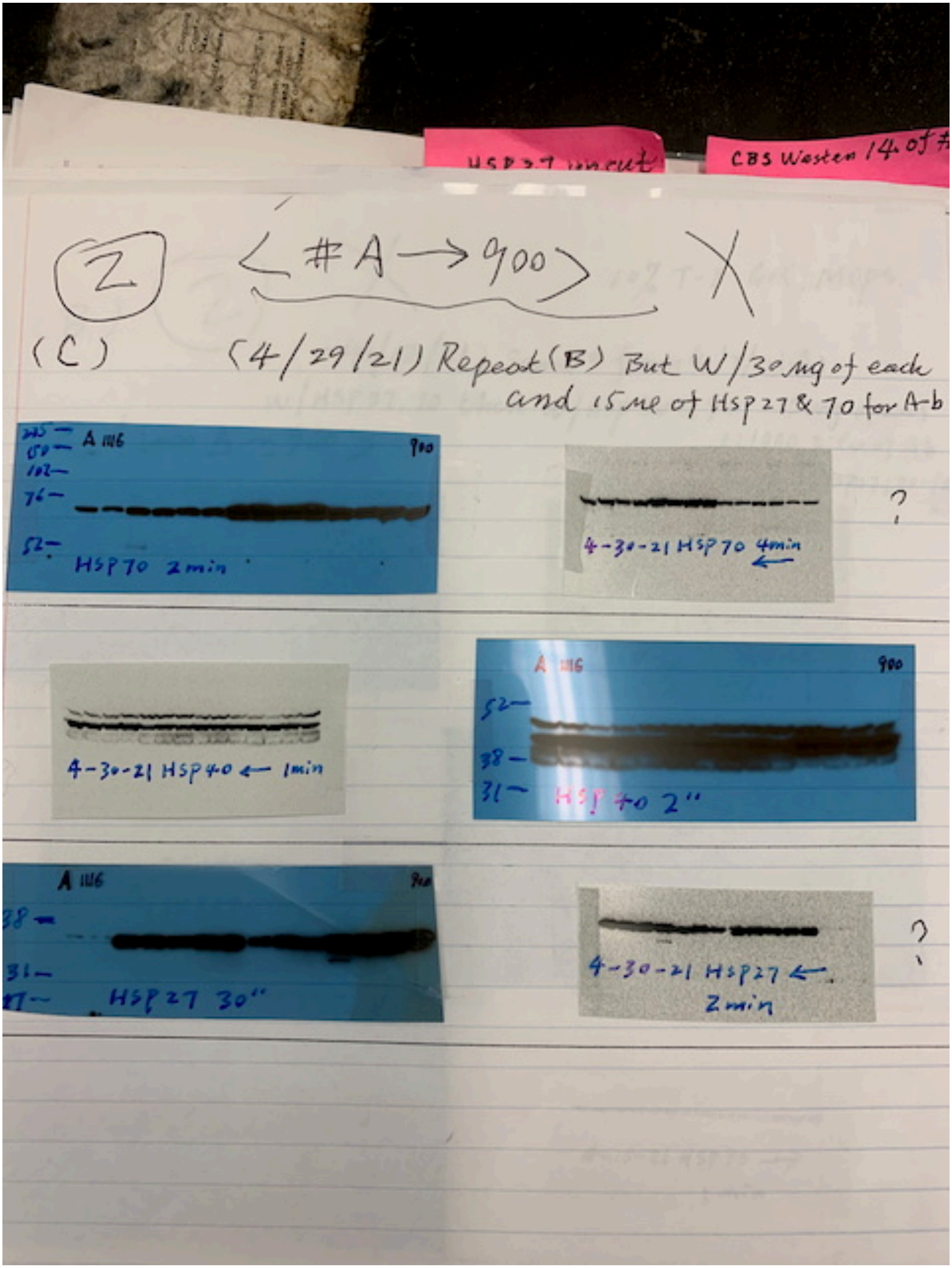

Figure 2c data

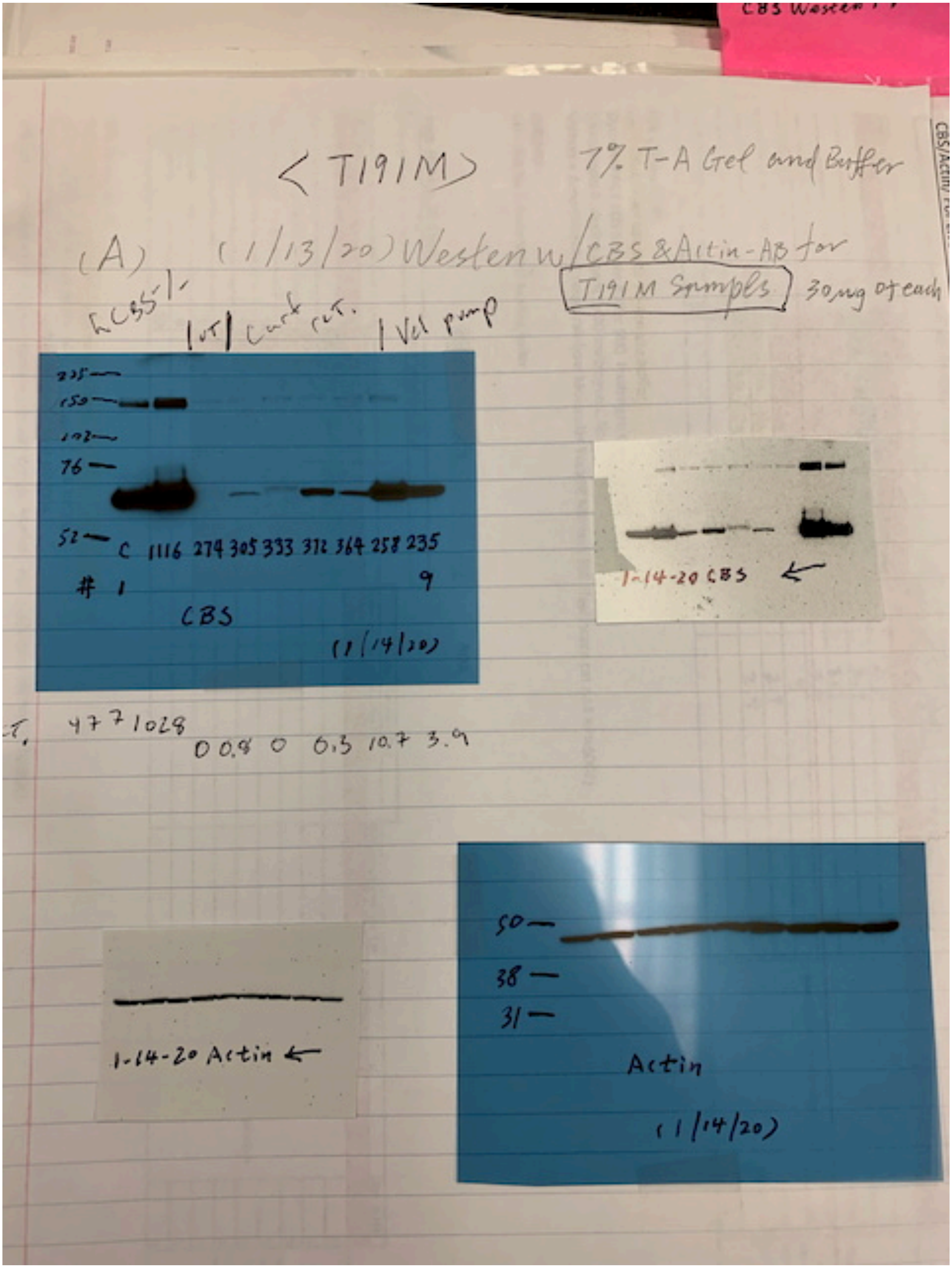

Figure 2D data.

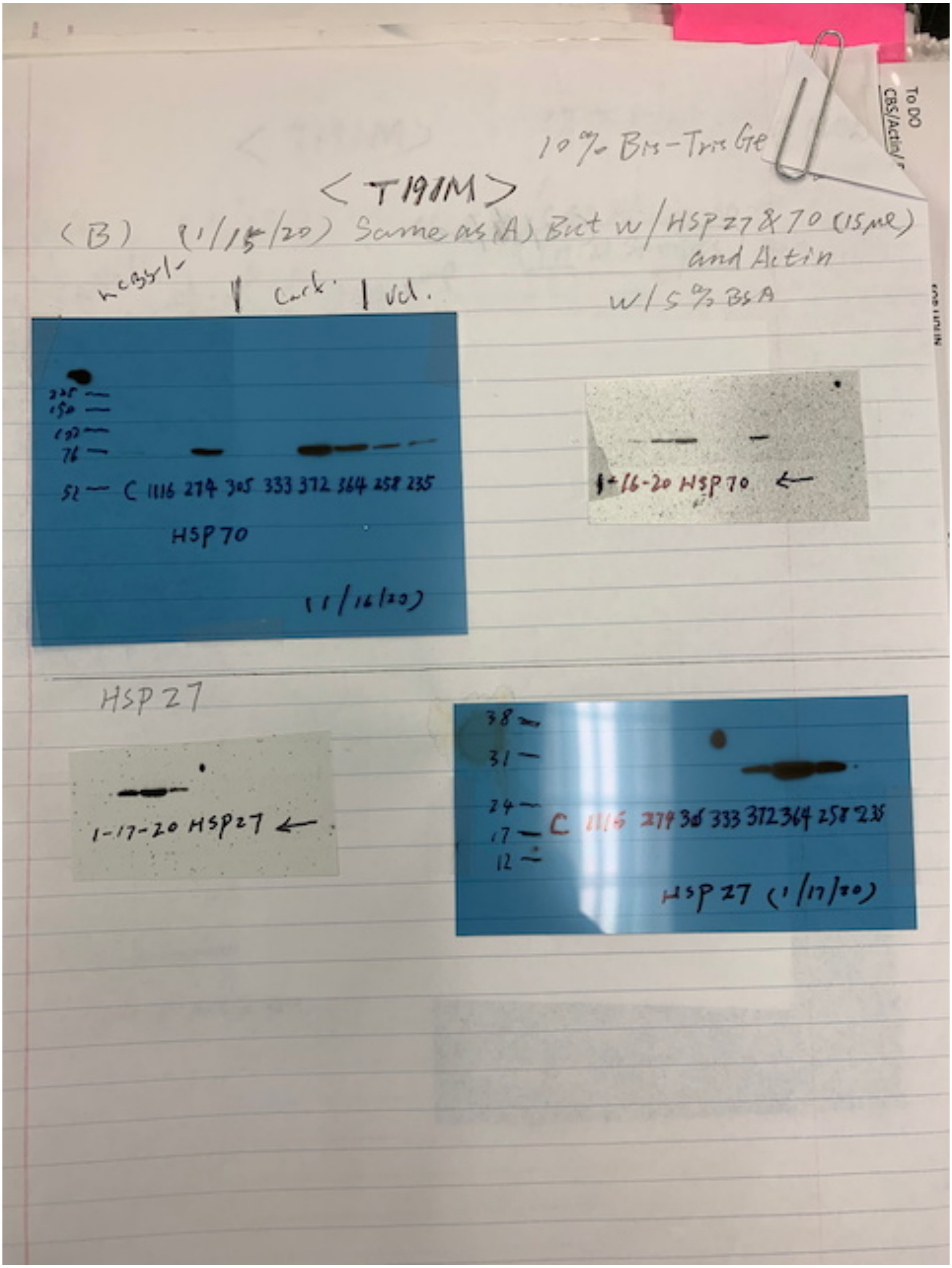

Figure 2d data

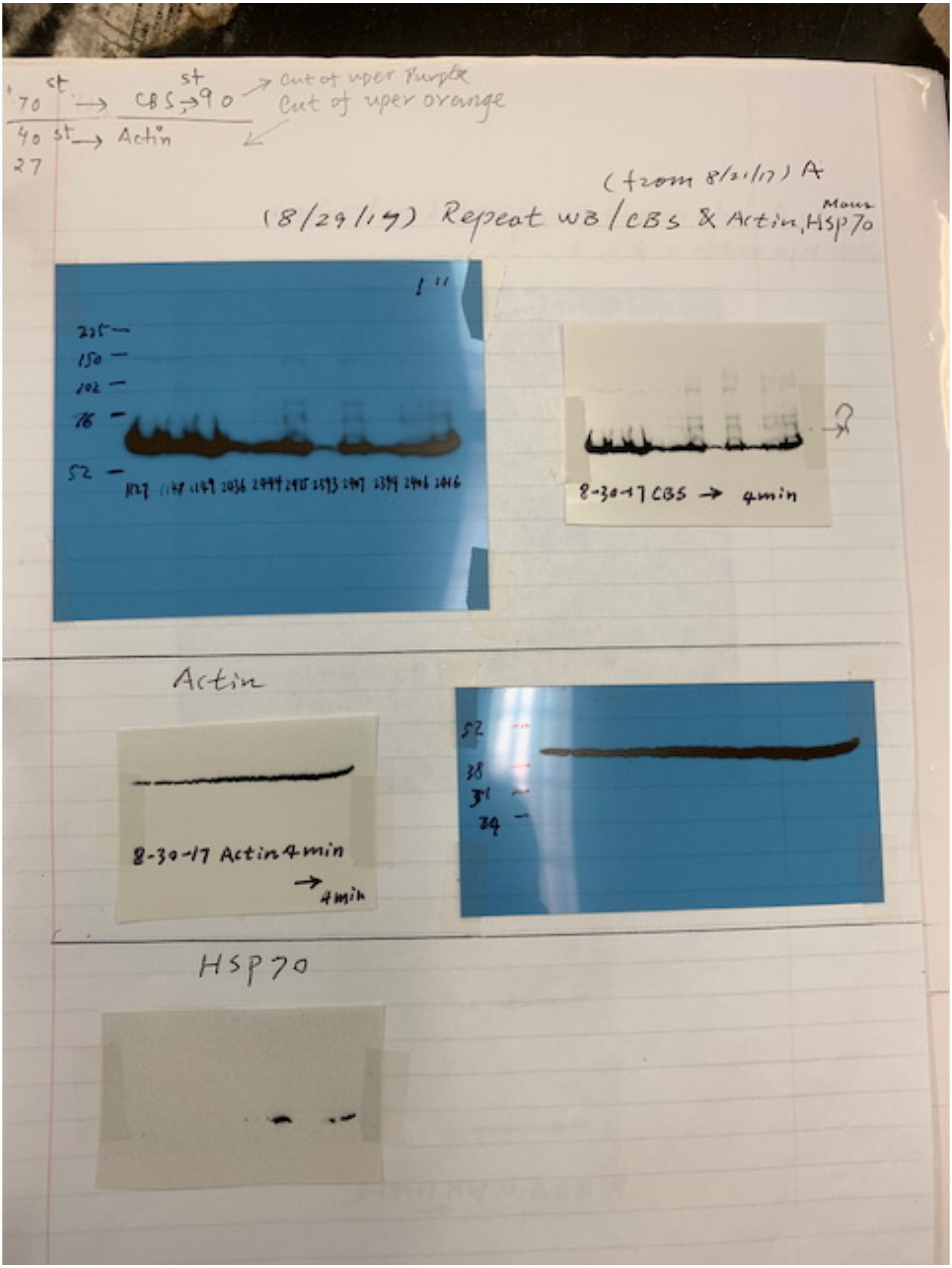
70 st → CBS, → 90 st
→ Cut of uper Purple
Cut of uper orange
40 st → Actin
27
(from 8/21/17) A
(8/29/14) Repeat WB / CBS & Actin, HSP70
Mous
225
150
102
76
52
8-30-17 CBS → 4min
Actin
8-30-17 Actin 4min
→ 4min
52
38
31
24
HSP70

Figure 6d

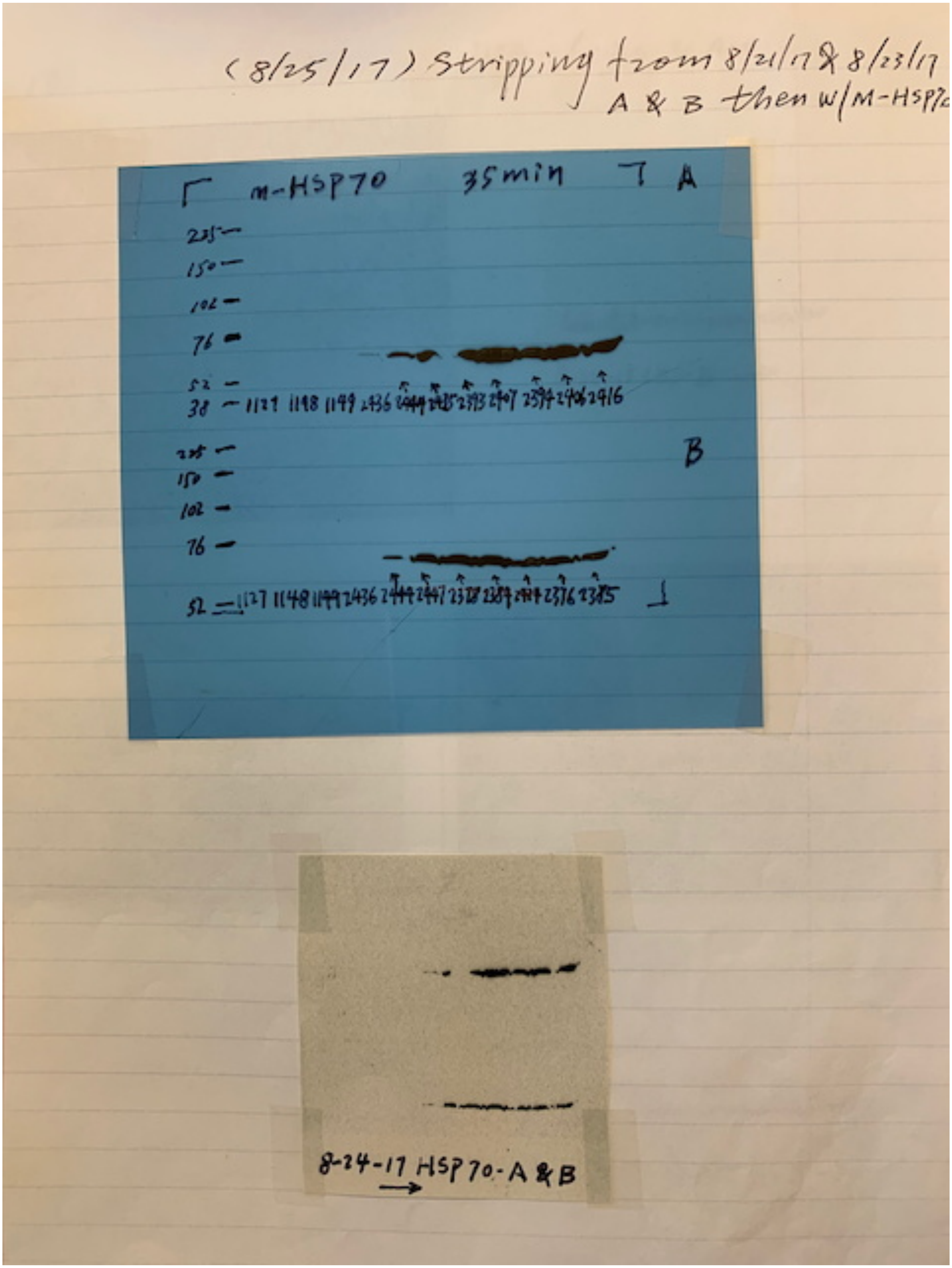
(8/25/17) Stripping from 8/21/17 & 8/23/17
A & B then w/M-HSP70
m-HSP70 35min A
B
8-24-17 HSP70-A & B

Figure 6d

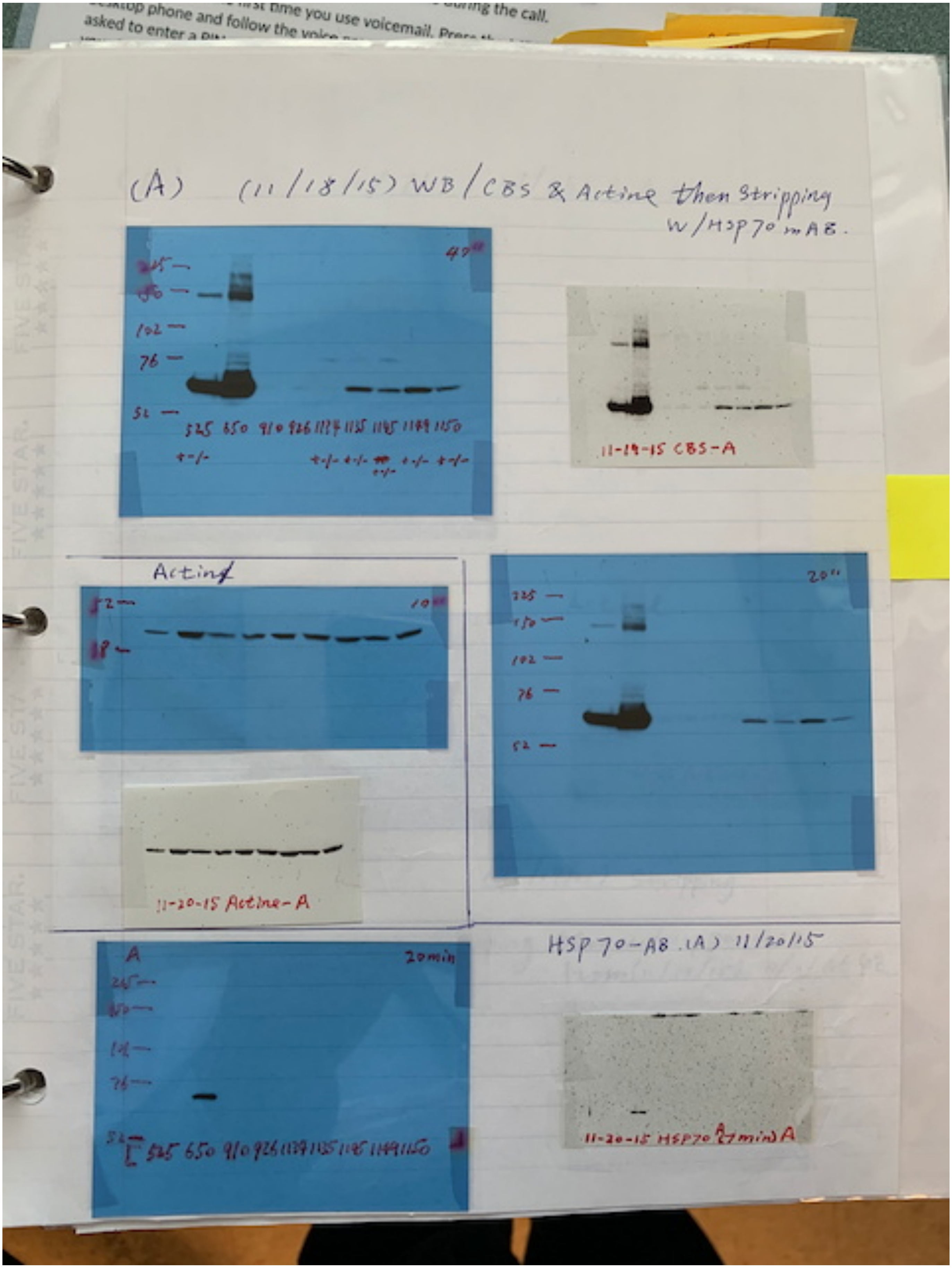

Figure 7C data

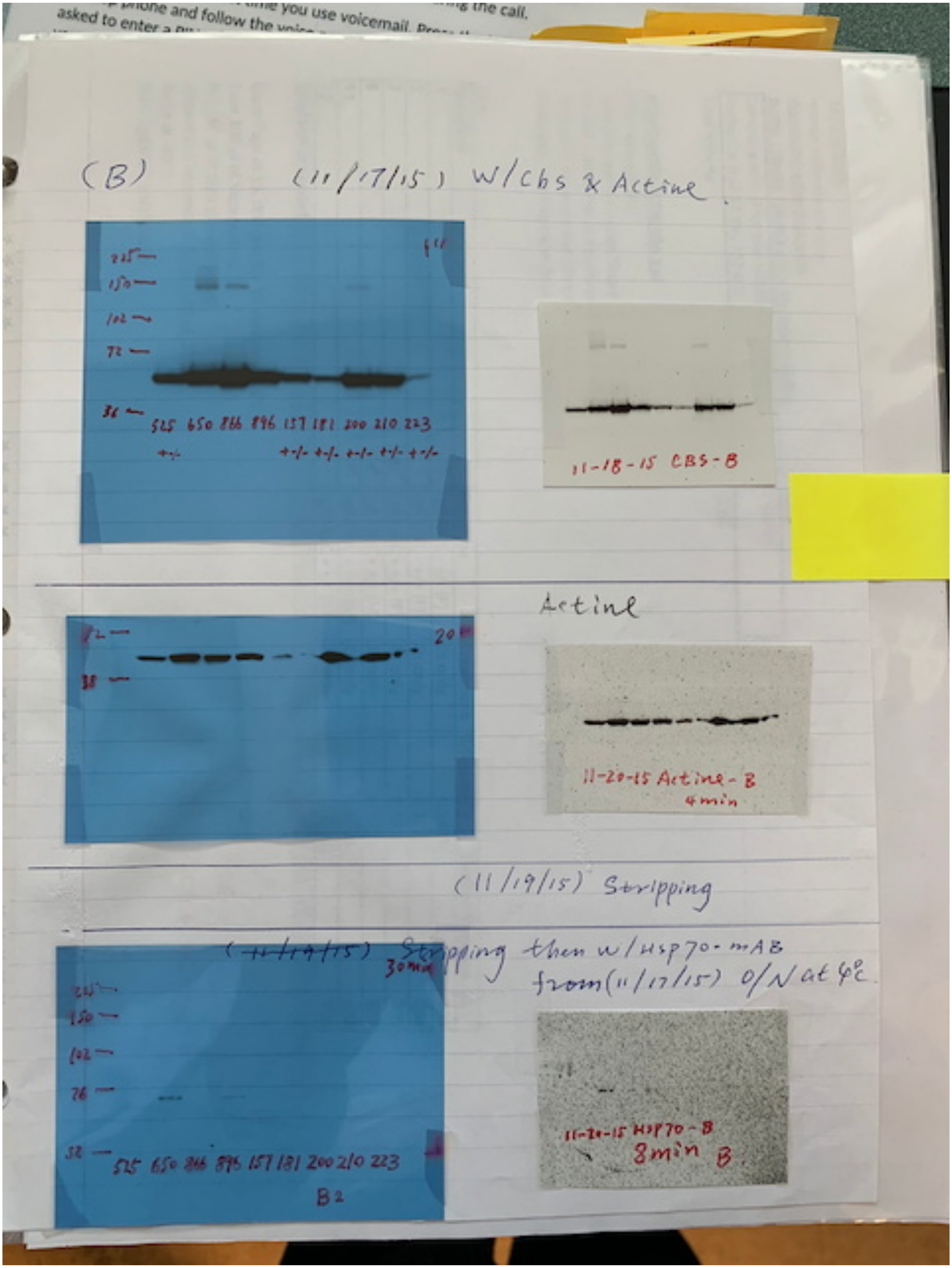

Figure 7C data
