# Supplementary material for: Examination of two different proteasome inhibitors in reactivating mutant human cystathionine β-synthase in mice
Source: PLoS One. 2023 Jun 15;18(6):e0286550. doi: 10.1371/journal.pone.0286550 (PMC10270616; doi:10.1371/journal.pone.0286550)
Supplement: Attachment — Submitted filename: Bortezomib vs. carfilzomib in CBS homocystinuria Review.docx [file pone.0286550.s003.docx]

Review of: “*Bortezomib is more effective than carfilzomib in reactivating mutant human cystathionine beta synthase in mice*”.

In this manuscript the authors investigate whether proteosome inhibition can restore expression of misfolded and/or unstable cystathionine beta synthase (CBS) protein, the absence of which cause homocystinuria. Homocystinuria manifests with elevated serum homocysteine levels in combination with diminished or absent CBS activity. Using a murine model, the authors compare the relative efficacy of two proteosome inhibitors, bortezomib and carfilzomib, in stabilizing and restoring the activity of defective CBS proteins with single amino acid substitutions, substitutions that otherwise compromise folding and lead to rapid proteosome-mediated degradation. While high doses of either drug restore some CBS activity and lower serum homocysteine levels, the authors conclude that bortezomib is more effective than carfilzomib. Interestingly, the authors find a poor correlation between proteosome activity—as measured by fluorogenic chymotrypsin—and drug efficacy, suggesting that other components of the protein regulation machinery must contribute to the approved therapeutic effects. Moreover, though both agents induce chaperone expression (hsp70 and hsp40)—which the authors interpret as a surrogate for protein refolding capacity—the induction is greater in carfilzomib, the less effective of the drugs. Unfortunately, both bortezomib and carfilzomib prove toxic to the mice, but lower doses prove neither effective nor resolve the toxicity of either agent, rending these drugs poor candidates for clinical use.

This is a well-written manuscript and the underlying work advances our understanding of the therapeutic potential of proteosome inhibitors. However, the authors should temper their conclusions, which at times oversimplify and overstate the findings. There are several specific areas that should be addressed:

1. The ability to draw specific conclusions regarding the relative efficacy of bortezomib and carfilzomib is limited by variability in the route of delivery and dose of the drugs. I appreciate that the authors extrapolated from the human chemotherapeutic dose. However, comparing retro-orbital injections to subcutaneous infusion introduces a great deal of uncertainty in pharmacodynamics and pharmacokinetics. As evidence of this concern, the author’s found that the effect of RO and SC carfilzomib differed in Tg-S466L animals, specifically with regard to the induction of Hsp70 and Hsp40. The authors do not address this confounding result. While the authors quantify fluorogenic chymotrypsin in the liver lysates as a surrogate for proteosome activity, they observe that there is poor correlation between fluorgenic chemotrypsin levels and CBS enzyme activity, suggesting that the effects of the drugs may be mediated—at least in part—by other mechanisms. *Given the available information—including factors addressed below—the authors would be better served to describe their findings as a class effect of proteosome inhibitors rather than to compare their relative efficacy of the two drugs.*
2. The authors do not adequately address why there is variability in proteasomal suppression between mouse strains (Figure 4) with the same genetic background. This finding would suggest inadequate numbers to properly evaluate, possible variability in drug delivery and/or issues with reliability of the assay.
3. Age and Sex Matching: The authors address whether the mice used in these experiments were age and sex matched. These variables profoundly affect animal physiology, and mismatching cohorts could compromise the conclusions drawn in this manuscript. The authors need to explicitly define the sex distribution and age-range of the animals.
4. I am also concerned about the limited number of animals assessed in various subgroups and experimental conditions, particularly those in which there is great variability within a subgroup/experimental condition. This is especially concerning if there is age/sex mismatching. Small cohorts include:
   a. Figure 1: 6 and 24 hour RO groups (panels A+ B).
   b. Figure 3: Carfilzomib-treatment groups (T191).
5. In the last sentence of the section entitled “Comparison of Carfilzomib and bortezomib rescue of various CBS mutations”, the author's state that carfilzomib is “not as effective as bortezomib in stimulating refolding of mutant CBS proteins into functional enzymes.” While function appears to be partially restored with drug treatment and effect may well be due to refolding, the authors present no direct evidence of this.
6. Figure 2: In 2C: The 4^th^ carfilzomib-treated animal has dramatically increased CBS expression compared to the others in the carfilzomib arm. Does this animal have different characteristics compared to the other 3? There is induction of HSP70 in hCBS animals while that phenomenon is not observed in any of the other panels (B and D) though the control is the same. This control is not included in panel A.
7. In the low dose bortezomib and/or carfilzomib experiments the authors missed an opportunity to treat their mutants with pyridoxine, which is a cofactor of CBS, and is known to have a therapeutic effect in at least R266K and I278T homozygous patients. While the authors previously showed that pyridoxine does not rescue CBS deficiency in mice when used alone, it could have an effect in the presence of a proteosome inhibitor—and potentially allow for lower doses of a proteosome inhibitor.
8. Minor typographical errors:
A. Page 4, lines 16-17 "involvement of other factors in restoration of mutant protein function apart only proteosome."
B. Page 8 line 15: "control animals =.
C. Page 10, line13-14: "__ small" "too" is missing.
D. Page 12, line 14: "addition of bortezomib". The authors presumably mean bimoclomol
E. Page 9: Line 11: "bortezomib did seem stimulate a very small".
