# Supplementary material for: Examination of two different proteasome inhibitors in reactivating mutant human cystathionine β-synthase in mice
Source: PLoS One. 2023 Jun 15;18(6):e0286550. doi: 10.1371/journal.pone.0286550 (PMC10270616; doi:10.1371/journal.pone.0286550)
Supplement: Attachment — Submitted filename: Response to Reviewers.docx [file pone.0286550.s004.docx]

Response to Reviewers:

We very much appreciate the Reviewers comments and will do our best to address them below.

Reviewer 1

*Given the available information—including factors addressed below—the authors would be better served to describe their findings as a class effect of proteosome inhibitors rather than to compare their relative efficacy of the two drugs.*

In response to the reviewers concerns, we have changed the title of the manuscript to read “Examination of two different proteasome inhibitors in reactivating mutant human cystathionine beta-synthase in mice”.

*The authors do not adequately address why there is variability in proteasomal suppression between mouse strains (Figure 4) with the same genetic background. This finding would suggest inadequate numbers to properly evaluate, possible variability in drug delivery and/or issues with reliability of the assay.*

We spent quite a bit of time optimizing the proteasome activity assay, performing several quality control measures, and found that it was quite consistent when the same sample was measured twice. The Reviewer is incorrect about the mice being the same genetic background. The transgenic founders are all made by injecting C57BL6/C3H F2 hybrids, and thus the founders can have different amounts of C3H and C57BL6 DNA. Without going into all the details, each line is then backcrossed two or three times to either to C57BL6 or C3H depending on whether the transgene suppresses the neonatal phenotype. (Please feel free to examine these papers for more of the details: PMID 15972722, 28488385, 31240737, 33949005). Thus, while each strain is mostly either C3H or BL6, there is still quite a bit of the other strain present. In addition, since each strain also expresses a different misfolded mutant CBS protein, we cannot rule out that it may also have an effect. Finally, It should be noted that the amount of variability observed in the proteasome activity assay is not much different than the variability we see in other biological measures such as plasma tHcy.

*Age and Sex Matching: The authors address whether the mice used in these experiments were age and sex matched. These variables profoundly affect animal physiology, and mismatching cohorts could compromise the conclusions drawn in this manuscript. The authors need to explicitly define the sex distribution and age-range of the animals.*

We have now included a supplemental file with information on the ages, sexes, tHcy, and CBS activity of the mice used in the study. Almost all of the mice in the study were females, as our previous work has shown that female mice have more consistent induction of the MT-I driven transgene that we use to make the mice (see PMID:15105297). The median age of the 138 mice used in the study was 5.6 months with a range of 2-25 months. We do not find that either sex or age has much of an effect of tHcy or CBS activity in humanized transgenic Cbs knockout mice.

*I am also concerned about the limited number of animals assessed in various subgroups and experimental conditions, particularly those in which there is great variability within a subgroup/experimental condition. This is especially concerning if there is age/sex mismatching. Small cohorts include:*

a. *Figure 1: 6 and 24 hour RO groups (panels A+ B).*
b. *Figure 3: Carfilzomib-treatment groups (T191).*

We wish these groups were larger as well. However, producing these mice is not trivial. *Cbs-/-* female mice lacking a WT human transgene cannot produce offspring. This means that we need to breed heterozygous transgene positive females to homozygous transgene positive males. The offspring from these crosses then need to be individually genotyped. Since we only use female mice for the studies, this means only ¼ mice are useful. On top of this, neonatal lethality is a problem in especially in the T191M line, so we had very few mice to put on trial. With regards to the RO panels in Figure 1, It was our first experiment trying carfilzomib in mice and the goal was just to get some hint of efficacy to decide if it warranted additional experiments. We feel the data in this regard is clear.

*In the last sentence of the section entitled “Comparison of Carfilzomib and bortezomib rescue of various CBS mutations”, the author's state that carfilzomib is “not as effective as bortezomib in stimulating refolding of mutant CBS proteins into functional enzymes.” While function appears to be partially restored with drug treatment and effect may well be due to refolding, the authors present no direct evidence of this.*

We have now changed the sentence to read: “Taken together, our results indicate that carfilzomib is not as effective as bortezomib in restoration of function to mutant CBS protein *in vivo.”*

*Figure 2: In 2C: The 4^th^ carfilzomib-treated animal has dramatically increased CBS expression compared to the others in the carfilzomib arm. Does this animal have different characteristics compared to the other 3? There is induction of HSP70 in hCBS animals while that phenomenon is not observed in any of the other panels (B and D) though the control is the same. This control is not included in panel A.*

The reviewer is correct. This animal (3008) has more enzyme activity and very low tHcys. We now point this out in the figure legend, and specifically point this out this animal in Figure 3. While I agree that there is some basal Hsp70 present in untreated hCBS mice and I278T mice, the levels are clearly lower than the bortezomib and carfilzomib treated ones. Since the hCBS samples are the same on all the blots, the background detection suggests that we had a bit more sensitivity or background on this particular blot for unknown reasons. The hCBS samples on blot B and D are the same exact samples that is on C, and those blots did not detect a Hsp70 signal.

*In the low dose bortezomib and/or carfilzomib experiments the authors missed an opportunity to treat their mutants with pyridoxine, which is a cofactor of CBS, and is known to have a therapeutic effect in at least R266K and I278T homozygous patients. While the authors previously showed that pyridoxine does not rescue CBS deficiency in mice when used alone, it could have an effect in the presence of a proteosome inhibitor—and potentially allow for lower doses of a proteosome inhibitor.*

- For the R266K mutation, we examined if liver extracts from bortezomib treated mice were more sensitive to PLP in the *in vitro* reaction mixture than wt CBS (see Figure 5B, PMID 28488385). Since this result was negative, we did not feel it was likely that the *in vivo* experiment would work. However, it would be an interesting experiment.

2. *Minor typographical errors:*
A. *Page 4, lines 16-17 "involvement of other factors in restoration of mutant protein function apart only proteosome."*

Fixed

B. *Page 8 line 15: "control animals =.*

Fixed

C. *Page 10, line13-14: "__ small" "too" is missing*.

Fixed

D. *Page 12, line 14: "addition of bortezomib". The authors presumably mean bimoclomol*

Fixed

E. *Page 9: Line 11: "bortezomib did seem stimulate a very small".*

*Reviewer #2: 

Largely as a description of comparative results I think the paper comes to appropriate conclusions, although I think the authors need to be even more explicitly cautious in their discussion. Not only does the dosing and route of administration potentially affect their outcomes, but so too does the fact that they are selecting only one time point for examination, and I could easily believe that the pharmacokinetics of the drug, combined with the synthesis and turnover of CBS, might yield different results if a more systematic examination of time points were conducted. The larger point of the paper, though, is that, despite the fact that it seems to inhibit the proteasome fairly robustly, CZ is not at least in these assays nearly as protective as BZ, which implies at least at face value that BZ works through proteasome-independent mechanisms. The authors mention this in the discussion, but I am a bit surprised that conclusion is not more front-and-center in the abstract.

Points for attention:
1. For CZ-treated animals were control mice injected and/or pumped (with water)? The methods do not indicate this, though they explicitly state that the BZ controls were pumped with diluent. If the controls for CZ were not so-treated, that would be in my opinion a pretty serious problem of rigor.*

The CZ-treated controls were a combination of mock treated (i.e. saline injected) and untreated animals. Specifically, we performed mock R.O. injections on a total of five animals (2 I278T, 1S466L, 1R266K and one R336C). We did not observe that any of mock treated mice had lowering of tHcy or increased CBS activity compared to the untreated animals. We have now added a new supplemental data that gives the specific information on every animal (including what controls were used) for each experiment.

*2. Related to the above, the experiment in Fig. 5 lacks (at least for the serum HCy and body weight) the control of animals injected with diluent on the same schedule. It is hard to interpret especially 5B without such a control. I can imagine that the stress of being repeatedly handled and jabbed might affect any number of physiological parameters. If that control wasn’t done, it either needs to be done or the experiment should not be included.*

The point of the experiments shown in Figure 5 and Figure 6 was to determine if low, non-toxic doses of bortezomib might lower tHcy like high doses do. While we did observe some modest lowering, from a pre-clinical perspective the data does not look very promising for human trials. We think this type of data is important to publish so others don’t waste their time. However, the reviewer is formally correct that it is possible that the modest lowering that was observed could be due to injection and handling. We have included this cavaet in the discussion section (p15 middle).

*3. Figure 6—it isn’t clear that the Bimo had any effects on the system at all. A simple explanation is that the compound is not active as intended. Absent a way to confirm that it is doing something in vivo, it’s a really difficult experiment to interpret and I would take it out.*

We agree that Bimo had no effect. However, the experiment confirms the repeatability of the lowering observed in Figure 5, so we decided to include it.

*4. The I278T animals are strange. CZ diminishes serum HCy but without any increase in CBS expression or activity. How do the authors account for this?*

In our experience, tHcy seems to be a more sensitive indicator of CBS activity than the in vitro assay. For example, the tHcy of Tg-hCBS Cbs-/- animals not on zinc is always lower than Tg-I278T Cbs-/- animals on zinc even though we cannot detect a difference in their liver CBS activity levels.

*5. Gels need migration standards.*

These are Western blots, not gels. Migration standards are marked on X-ray film with marker after lining up the blot. We have included images of the actual X-ray film in the data supplemental.

*6. Label font sizes in Fig. 2 are discrepant and the correlation graphs in Fig. 4 look like they were dropped straight from Excel. Showing treated animals to the left in 5E is a bit unconventional, and I would recommend keeping color designations (UT, BZ, CZ) consistent in Fig. 3. Overall for aesthetics I think the figures should be cleaned up.*

We re did the correlation graphs in Prism.
