# Supplementary material for: Examination of two different proteasome inhibitors in reactivating mutant human cystathionine β-synthase in mice
Source: PLoS One. 2023 Jun 15;18(6):e0286550. doi: 10.1371/journal.pone.0286550 (PMC10270616; doi:10.1371/journal.pone.0286550)
Supplement: Attachment — Submitted filename: Repsonse to Reviewer.docx [file pone.0286550.s005.docx]

*1. Please edit/clarify the following added text, "in inducing in inducing", and "that the lowering..."*

Fixed

*2. Please add some additional details to the construction of the mouse strain in the Methods, with regard to Reviewer 1's comment on the background.*

Added details in Methods section.

*3. Please be specific in the text with regard to "almost all the mice"...numbers would be helpful here.*

Added percentage of mice that were female.

4*. Based on the suggested title change, please add a sentence or two placing your work in the context of a prior paper on the effects of different proteasome inhibitors in another conformational disease (PMID: 18775310)*

Added sentence referencing this paper in introduction.
